# Inhibition of Fibroblast Growth Factor Receptor 3 Signaling by Ponatinib Reduces Growth and Cytokine Production of Multiple Myeloma Cells

**DOI:** 10.3390/ijms27125217

**Published:** 2026-06-09

**Authors:** Sascha Kampmann, Sebastian Schlaweck, Benjamin V. Becker, Chrystel Flores, Annkristin Heine, Peter Brossart, Stefanie A. E. Held

**Affiliations:** 1 Department of Hematology, Oncology, Stem Cell Transplantation, Immune and Cell Therapy, Clinical Immunology and Rheumatology, University Hospital Bonn, 53127 Bonn, Germany; sascha.kampmann@ukbonn.de (S.K.); sebastian.schlaweck@ukbonn.de (S.S.); peter.brossart@ukbonn.de (P.B.); 2 Center for Integrated Oncology Aachen Bonn Cologne Düsseldorf (CIO ABCD), 53127 Bonn, Germany; 3 Mildred Scheel School of Oncology Aachen Bonn Cologne Düsseldorf (MSSO ABCD), Faculty of Medicine, University Hospital of Bonn, 53127 Bonn, Germany; 4 Department of Radiology and Neuroradiology, Bundeswehr Central Hospital, 56072 Koblenz, Germany; 5 Division of Hematology, Medical University of Graz, 8036 Graz, Austria

**Keywords:** multiple myeloma, FGFR3, ponatinib, targeted therapy

## Abstract

Recurrent genetic and chromosomal aberrations drive multiple myeloma (MM) pathogenesis. Among these, the t(4;14) translocation leads to overexpression of fibroblast growth factor receptor 3 (FGFR3) and is associated with poor prognosis. However, therapeutic approaches directly targeting FGFR3-driven myeloma progression remain limited. Here, we investigated the single-agent activity of ponatinib, a multikinase inhibitor, in MM. KMS18 and U266 myeloma cell lines were treated with ponatinib, and apoptosis induction, as well as VEGF and IL-6 secretion, was assessed. RNA sequencing of MM cells revealed pathway alterations induced by ponatinib treatment, which were subsequently validated by Western blot analysis. In vivo, mice inoculated with 5T33 myeloma cells received ponatinib, and survival was monitored. Notably, ponatinib exerted potent single-agent antimyeloma activity in an FGFR3-dependent manner by inducing apoptosis and suppressing VEGF and IL-6 secretion through inhibition of JAK/STAT, PI3K/AKT, and MAPK signaling. In vivo administration prolonged survival in myeloma-bearing mice. Collectively, our findings demonstrate the therapeutic efficacy of ponatinib in FGFR3-expressing MM beyond selective FGFR3 inhibition, suggesting that concurrent suppression of multiple signaling pathways is a critical mechanism of action. These results highlight the therapeutic potential of combined FGFR3-targeted strategies in multiple myeloma and provide a rationale for further clinical investigation.

## 1. Introduction

Multiple myeloma (MM) is an incurable plasma cell neoplasia characterized by exceptional heterogeneity. The survival, but also treatment responses, of MM patients depend on molecular characteristics. High-risk patients with poor prognosis may be identified by genomic or chromosomal aberrations [1]. Approaches with whole-genome/exome and RNA sequencing have identified different subgroups, in which almost 5% of newly diagnosed patients with MM harbor mutations in the fibroblastic growth factor receptor 3 (*FGFR3*) gene [2]. *FGFR3* gene mutations are significantly increased in MM cells with a chromosomal translocation between chromosomes 4 and 14 (t(4;14) [3], which is phenotypically characterized by FGFR overexpression [4]. Overall, in approximately 13% of all myeloma patients, including newly diagnosed and refractory/relapsed patients, this translocation is observed [5]. Notably, the *FGFR3* gene was found to be predominantly mutated in genetically high-risk patients [6], whereas functionally high-risk patients harbor mutations in genes affecting interleukin 6 (IL-6); Janus kinase (JAK) and signal transducer and activator of transcription 3 (STAT3) signaling; glycolysis; hypoxia tolerance; and oxidative stress, as well as DNA damage repair pathways [1]. Especially in combination with concomitant aberrations, such as chromosome 1q gain/amplification, t(4;14) is associated with worse outcomes [7].

FGFR signaling has pleiotropic functions in health and disease [8], including activation of inflammatory pathways and angiogenesis, which are also hallmarks of myeloma progression [9,10]. FGFR3 activation causes continuous stimulation of different pathways, including the following: mitogen-activated protein kinase (RAS-RAF-MAPK), phosphatidylinositol 3-kinase (PI3K-AKT-mTOR), phospholipase Cγ (PLCγ), protein kinase C (PKC), and STAT signaling [5]. In MM, this is accompanied by the proliferation of malignant cells and clinical disease progression [11].

Although the implication of proteasome inhibitors [12] and immunomodulatory drugs (IMiD) [13] in treatment regimens has improved the limited outcomes of patients with t(4;14), subsequent therapies with conventional regimens have yielded only low and non-durable response rates [14] in triple class-exposed patients. However, novel T cell-directed therapies, which revolutionized MM treatment, target B cell maturation antigen (BCMA) and glycoprotein receptor C5D (GPRC5D) [15,16,17], but not FGFR3. Early approaches inhibiting FGFR3 signaling showed preclinical activity 20 years ago [18]. Erdafitinib, a small molecule inhibiting FGFR 1–4, was investigated in relapsed/refractory MM (NCT02952573). Although final results have not been reported, a case report highlights that targeted treatment might eradicate the FGFR3-mutated clone [19]. Therefore, interfering with the FGFR3 pathway may be a promising therapeutic target in myeloma.

Ponatinib is approved for the treatment of chronic myeloid leukemia and acute lymphatic leukemia with bcr-abl fusion and has shown impressive response rates in Philadelphia-positive leukemias [20]. Moreover, ponatinib has been identified as a pan-FGFR inhibitor [21]. The efficacy of ponatinib combination regimens in preclinical models of MM has already been described. Ponatinib, either in combination with mitogen-activated protein kinase kinase (MEK) [22] or mammalian target of rapamycin (mTOR) [23] inhibition, is capable of killing malignant plasma cells. In the first study, ponatinib and trametinib prolonged myeloma-dependent survival in mouse experiments, while in the latter, the combination regimen of ponatinib + sirolimus inhibited oxidative phosphorylation, leading to myeloma cell death.

Although these studies have already identified ponatinib as a potential novel drug for combination therapies in MM treatment, molecular characterization of its mode of action is lacking. Furthermore, the single-agent activity of ponatinib was not investigated in detail.

Therefore, we sought to unveil the molecular pathways altered by ponatinib treatment in MM with a special focus on FGFR3 pathway alterations.

## 2. Results

### 2.1. Ponatinib Dampens the Expression and Phosphorylation of FGFR3 on the Surface of KMS18 Myeloma Cells

**Figure 1:**
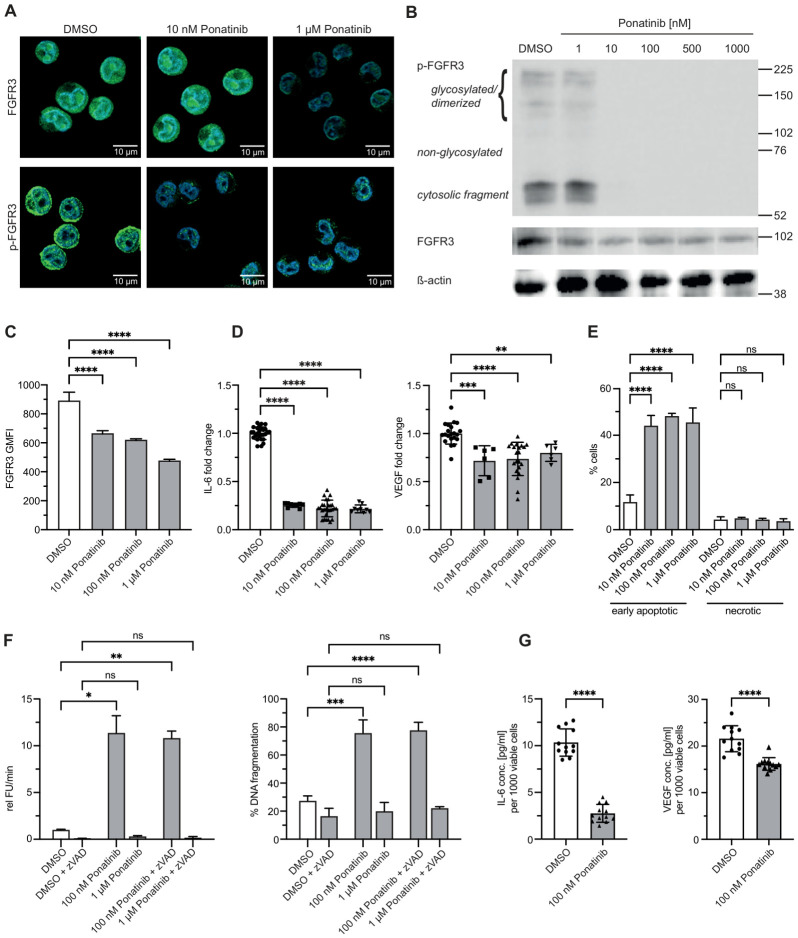
Ponatinib impairs FGFR3 expression and cytokine production and leads to caspase-dependent apoptosis in KMS18 cells. KMS18 cells were treated with ponatinib in the indicated concentrations or vehicle (dimethyl sulfoxide (DMSO)) for 24 h (**A**,**C**–**G**) or 1 h (**B**). (**A**) Merged confocal images showing FGFR3 or phosphorylated FGFR3 (p-FGFR3) (green) and nuclei (DAPI, blue) in KMS18 cells cultured on poly-L-lysine-coated coverslips. Scale bar, 10 µm. Data are representative of *n* = 3 independent experiments. (**B**) Representative immunoblots showing total FGFR3, p-FGFR3 and β-actin (loading control) in KMS18 cell lysates. Molecular weight markers (kDa) are shown. Data are representative of at least *n* = 2 independent experiments. (**C**) Flow cytometry analysis of surface FGFR3 geometric mean fluorescence intensities (GMFI) on live KMS18 cells. Pooled data from *n* = 3 independent experiments; mean ± SD (one-way ANOVA vs. vehicle). (**D**) ELISA quantification of interleukin 6 (IL-6) and vascular endothelial growth factor (VEGF) in culture supernatants from KMS18 cells. Data normalized to the vehicle control; pooled data from at least *n* = 9 (IL-6) or *n* = 6 (VEGF) independent experiments; normalized to vehicle mean ± SD (one-way ANOVA vs. vehicle). (**E**) Flow cytometry analysis of apoptosis and necrosis using Annexin V/7-aminoactinomycin D (7AAD) staining. Annexin V+/7AAD− cells represent early apoptotic cells; Annexin V±/7AAD+ cells represent late apoptotic/necrotic cells. Pooled data from *n* = 3 independent experiments; mean ± SD (one-way ANOVA vs. vehicle). (**F**) Fluorogenic caspase activity assay in KMS18 cell lysates following ponatinib treatment. DNA fragmentation was measured using propidium iodide (PI) staining. Pooled data from *n* = 3 (caspase activity) or *n* = 6 (PI staining) independent experiments; mean ± SD (one-way ANOVA vs. vehicle ± Z-VAD-FMK caspase inhibitor (zVAD)). (**G**) ELISA quantification of IL-6 and VEGF in culture supernatants from KMS18. Supernatants were collected for ELISA, and corresponding cell pellets were analyzed for live cells using Annexin V/ 7AAD staining. Data normalized to surviving cell number; pooled data from *n* = 12 independent experiments; mean ± SD (one-way ANOVA vs. vehicle). **** *p* < 0.0001, *** *p* < 0.001, ** *p* < 0.01, * *p* < 0.05, ns *p* ≥ 0.05.

The KMS18 cell line is a human multiple myeloma cell line harboring a G384D mutation in the *FGFR3* gene, which results in ligand-independent receptor phosphorylation. To assess FGFR3 expression and activation status, fluorescence microscopy was used to analyze total FGFR3 and phosphorylated FGFR3 (p-FGFR3) in KMS18 cells in vitro. Baseline analyses revealed substantial phosphorylation of FGFR3 in untreated cells. In contrast, treatment with ponatinib led to a dose-dependent reduction in pFGFR3 expression at the cell surface (Figure 1A). These findings were corroborated by Western blot analyses, which confirmed a dose-dependent decrease in FGFR3 phosphorylation upon ponatinib exposure (Figure 1B). In addition, flow cytometric analysis demonstrated an overall reduction in surface FGFR3 expression following ponatinib treatment (Figure 1C).

### 2.2. IL-6 and VEGF Levels Are Reduced by Ponatinib Treatment

Having demonstrated that ponatinib inhibits FGFR3 phosphorylation in myeloma cells, we next assessed downstream functional effects by analyzing cytokines known to be regulated by FGFR signaling [8] and relevant for myeloma growth, survival, and disease progression. Treatment with ponatinib resulted in a pronounced, dose-dependent reduction in IL-6 and VEGF levels. Notably, significant downregulation was already observed at the lowest tested concentration of 10 nM (Figure 1D).

### 2.3. Ponatinib Induces Apoptosis in Multiple Myeloma Cell Lines

Next, we wondered whether reduced cytokine levels are associated with apoptosis in myeloma cells. Therefore, we investigated the dose-dependent effect of ponatinib on apoptosis and necrosis of KMS18 cells in vitro. In detail, we showed, by staining for 7AAD and Annexin V, that ponatinib, as a single agent, selectively induces apoptosis. In sharp contrast, after an incubation period of 24 h, ponatinib treatment did not induce necrosis, implicating a rather specific effect on cellular signaling pathways (Figure 1E). Consistent with these observations, we demonstrated that ponatinib-induced apoptosis induction is caspase-3-dependent, as treatment with the pan-caspase inhibitor zVAD effectively abolishes ponatinib-mediated cell death (Figure 1F). However, IL-6 and VEGF levels, normalized to surviving MM cells, remained significantly decreased, suggesting the pleiotropic effects of ponatinib treatment (Figure 1G).

### 2.4. RNA Sequencing of Ponatinib-Treated KMS18 Cells Identifies Deregulated Oncogenic Pathways Central to Multiple Myeloma Progression, Confirmed by Proteomic Validation

**Figure 2:**
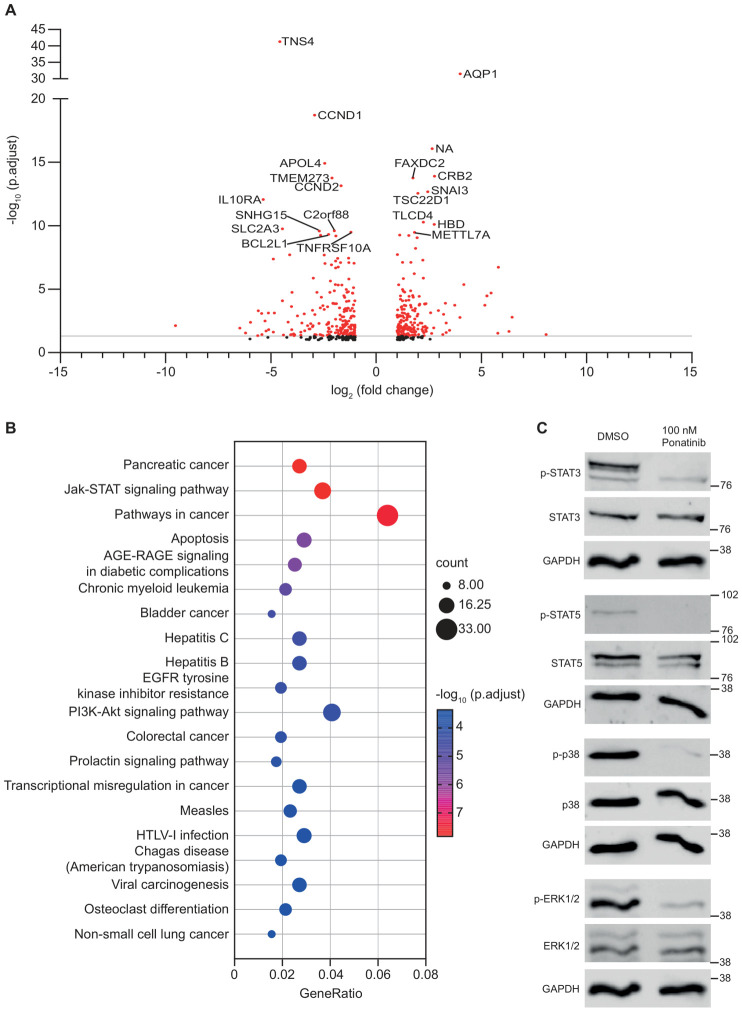
Ponatinib treatment deregulates oncogenic pathways central to MM progression in KMS18 cells. (**A**) Volcano plot of differentially expressed genes (DEGs) in KMS18 cells treated with 100 nM ponatinib vs. vehicle control (dimethyl sulfoxide (DMSO)) for 6 h. Red points: significantly deregulated DEGs (log2FC ≤ −1 or log2FC ≥ 1, adjusted *p* < 0.05); black points: non-significant. *n* = 1 with three technical replicates per condition; *p*-values adjusted for multiple testing (Benjamini–Hochberg). Top 20 DEGs labeled. (**B**) Kyoto Encyclopedia of Genes and Genomes (KEGG) pathway enrichment analysis of the abovementioned DEGs from RNA-sequencing of KMS18. Dot plot showing the top 20 enriched pathways ranked by adjusted *p*-value. Dot size = gene count; dot color = −log_10_(p.adjust). Full pathway list in Table 1. (**C**) Representative immunoblots showing total STAT3, STAT5, p38, ERK1/2, their corresponding phosphorylated forms and GAPDH (loading control) in KMS18 cell lysates. Cells were treated with 100 nM ponatinib or DMSO for 1 h. Molecular weight markers (kDa) are shown. Data are representative of at least *n* = 2 independent experiments.

To reveal the pathways altered by ponatinib-treatment other than FGFR, we applied RNA sequencing of ponatinib- and DMSO-treated KMS18 cells and identified more than 408 genes that were significantly dysregulated. Most strikingly, expression of the following genes was altered: *TNS4*, *AQP1*, *CCND1*, *APOL4*, *CRB2*, *TMEM273*, *FAXDC2*, *CCND2*, *SNAI3*, *TSC22D1*, *IL10RA*, *TLCD4*, *HBD*, *SLC2A3*, *C2orf88*, *SNHG15*, *TNFRSF10A*, *METTL7A*, and *BCL2L1* (Figure 2A).

KEGG pathway analysis of ponatinib-treated KMS18 cells was performed, and our results indicate a pleiotropic effect of ponatinib in MM treatment. Pathways associated with cytokine production—like JAK/STAT, PI3K/AKT, tumor necrosis factor (TNF), MAPK, and nuclear factor kappa-light-chain-enhancer of activated B cell (NFKB) signaling, but also cell death, such as forkhead box O (FoxO), apoptosis and tumor protein 53 (p53) signaling—were dysregulated (Figure 2B, Table 1). To confirm our results, we assessed the phosphorylation of key signaling proteins in ponatinib-treated KMS18 cells. Ponatinib treatment markedly reduced the phosphorylation of p38 and ERK1/2 MAPKs, as well as STAT3 and STAT5, which was consistent with the dysregulated pathways identified in the transcriptomic analysis (Figure 2C).

### 2.5. Selective FGFR3 Inhibition Does Not Induce Apoptosis but Dampens IL-6 and VEGF Secretion

**Figure 3:**
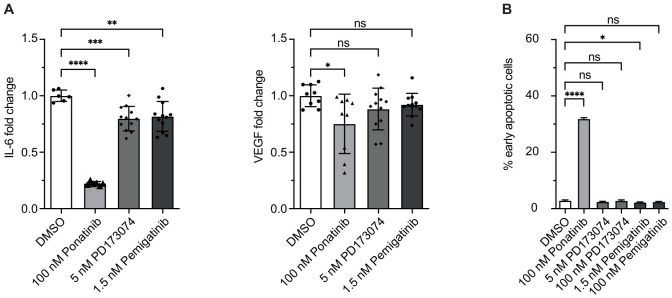
The FGFR3 inhibitors PD173074 and pemigatinib reduce cytokine production but do not induce apoptosis. KMS18 cells were treated with ponatinib, PD173074 or pemigatinib in the indicated concentrations or vehicle (dimethyl sulfoxide (DMSO)) for 24 h. (**A**) ELISA quantification of interleukin 6 (IL-6) and vascular endothelial growth factor (VEGF) in culture supernatants from KMS18 cells. Data normalized to the vehicle control; pooled data from at least *n* = 6 (IL-6) or *n* = 9 (VEGF) independent experiments; normalized to vehicle mean ± SD (one-way ANOVA vs. vehicle). (**B**) Flow cytometry analysis of apoptosis using Annexin V/7-aminoactinomycin D (7AAD) staining. Annexin V+/7AAD− cells represent early apoptotic cells. Pooled data from *n* = 3 independent experiments; mean ± SD (one-way ANOVA vs. vehicle). **** *p* < 0.0001, *** *p* < 0.001, ** *p* < 0.01, * *p* < 0.05, ns *p* ≥ 0.05.

As FGFR signaling controls cell survival, proliferation and angiogenesis by activating downstream kinases [8], we wondered whether selective FGFR3 inhibition accounts for the observed effects. Therefore, we evaluated the impact of PD173074 and pemigatinib, two different selective FGFR3 inhibitors, on IL-6 and VEGF secretion and apoptosis induction in KMS18 cells. Both inhibitors reduced IL-6 secretion dose-dependently and modestly suppressed VEGF release (Figure 3A), but neither triggered apoptosis, even in the highest concentrations up to 100 nM (Figure 3B).

### 2.6. The Single-Agent Activity of Ponatinib Depends on FGFR3

**Figure 4:**
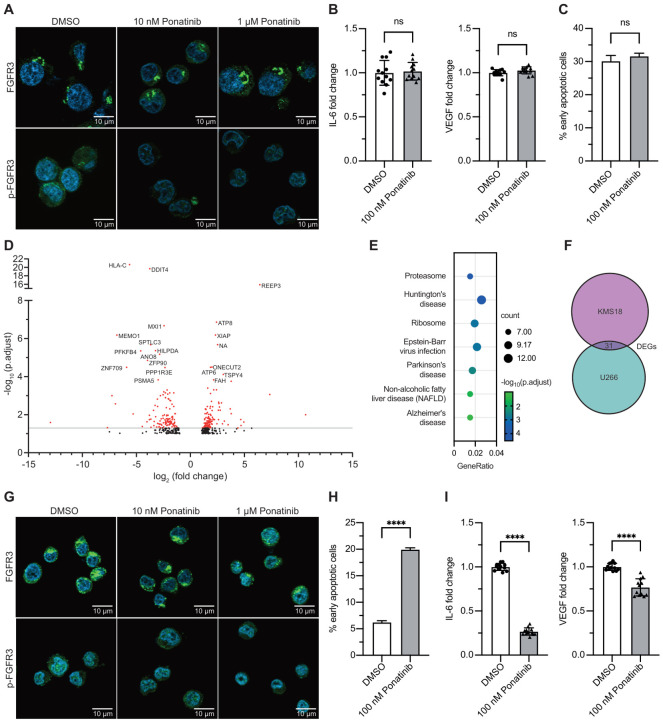
Ponatinib does not influence cytokine production or apoptosis in the surface FGFR3-lacking cell line U266. U266 cells were treated with ponatinib in the indicated concentrations or vehicle (dimethyl sulfoxide (DMSO)) for 24 h (**A**–**C**) or 6 h (**D**–**F**). MM.1S cells were treated with ponatinib in the indicated concentrations or vehicle (DMSO) for 24 h (**G**–**I**). (**A**) U266 cells cultured on poly-L-lysine-coated coverslips and stained for FGFR3 or phosphorylated FGFR3 (p-FGFR3) (green) and nuclei (DAPI, blue). Scale bar, 10 µm. Three independent experiments. (**B**) Interleukin 6 (IL-6) and vascular endothelial growth factor (VEGF) measured in culture supernatants from U266 cells via ELISA; 12 independent experiments; normalized to vehicle mean ± SD (one-way ANOVA). (**C**) Annexin V+/7-aminoactinomycin D (7AAD)- cells represent early apoptotic cells, analyzed by flow cytometry. Three independent experiments; mean ± SD (one-way ANOVA). (**D**) Volcano plot of differentially expressed genes (DEGs) in U266 cells treated with 100 nM ponatinib vs. vehicle control (DMSO). Red points: significantly deregulated DEGs (log2FC ≤ −1 or log2FC ≥ 1, adjusted *p* < 0.05); black points: non-significant. *n* = 1 with three technical replicates per condition; *p*-values adjusted for multiple testing (Benjamini–Hochberg). Top 20 DEGs labeled. (**E**) Kyoto Encyclopedia of Genes and Genomes (KEGG) pathway enrichment analysis of the abovementioned DEGs. Dot plot showing enriched pathways ranked by adjusted *p*-value. Dot size = gene count; dot color = −log_10_(p.adjust). (**F**) Venn diagram showing overlap of 31 DEGs (log2FC ≤ −1 or log2FC ≥ 1, adjusted *p* < 0.05) in KMS18 and U266 cells. *n* = 1; three technical replicates per condition. (**G**) MM.1S cells cultured on poly-L-lysine-coated coverslips stained for FGFR3 or p-FGFR3 (green) and nuclei (DAPI, blue). Scale bar, 10 µm. Three independent experiments. (**H**) Annexin V+/7AAD− cells, analyzed by flow cytometry, represent early apoptotic cells. Three independent experiments; mean ± SD (one-way ANOVA). (**I**) ELISA quantification of IL-6 and VEGF in culture supernatants from MM.1S cells. Data normalized to vehicle control; 12 independent experiments; normalized to vehicle mean ± SD (one-way ANOVA). **** *p* < 0.0001, ns *p* ≥ 0.05.

The human MM cell line U266 is characterized by dependence on an autocrine IL-6 signaling loop and consecutive STAT3 activation [24]. Here we show only very low and almost exclusively intracellular FGFR3 expression and phosphorylation in this cell line (Figure 4A). Interestingly, this was accompanied by unchanged IL-6 and VEGF levels after ponatinib treatment (Figure 4B). Consistent with these observations, ponatinib was also unable to induce apoptosis under these conditions (Figure 4C).

For mechanistic insight, again, we performed transcriptomic profiling following ponatinib exposure. In U226 cells, 242 significantly regulated transcripts were identified, fewer than those identified in KMS18 cells (Figure 4D). Subsequent KEGG pathway enrichment revealed perturbations in proteasome and ribosome function (Figure 4E). Finally, comparing the transcriptional responses of KMS18 and U266 cells to ponatinib treatment, we identified only 31 overlapping differentially expressed genes (Figure 4F).

As we have shown differences in the response of MM cells to ponatinib comparing a cell line with almost absent surface FGFR3 expression (U266) and overexpressed FGFR3 (KMS18), we next investigated the effect of ponatinib on MM.1S cells. MM.1S expressed FGFR3 in a wild-type-like manner and, due to the absence of the t(4;14) translocation, only very low levels of p-FGFR3. Ponatinib treatment reduced FGFR3 expression (Figure 4G) and induced apoptosis in vitro (Figure 4H), but on a smaller scale compared to KMS18 cells. Moreover, ponatinib treatment results in a concomitant reduction in IL-6 and VEGF levels (Figure 4I).

### 2.7. Myeloma-Dependent Survival Is Prolonged in Ponatinib-Treated Animals

**Figure 5:**
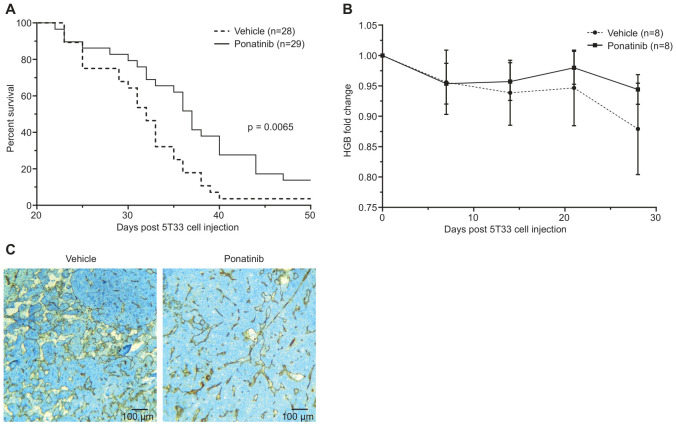
Ponatinib treatment prolongs survival and reduces neo-angiogenesis in the 5T33/KaLwRij MM mouse model. (**A**) Kaplan–Meier survival curves of C57BL6/KaLwRij mice injected intravenously (i.v.) with 5T33 MM cells (3 × 10^5^/mouse). Mice were treated with ponatinib (30 mg/kg) or vehicle control (25 mM citrate buffer) by daily oral gavage, starting at day 14 post-cell injection with a maximum endpoint at day 50. Vehicle (*n* = 28, 1 censored, median 32 days [95% confidence interval (CI) 29–33]); ponatinib (*n* = 29, 4 censored, median 37 days [95% CI 32–40]). Log-rank (Mantel–Cox) test: *p* = 0.0065. Hazard ratio (HR) (ponatinib/vehicle) = 0.42 (95% CI 0.23–0.79). (**B**) Frequency of hemoglobin (HGB) concentration in the mouse blood of C57BL6/KaLwRij mice injected i.v. with 5T33 MM cells (3 × 10^5^/mouse). Mice (*n* = 8–9 per group) were treated as mentioned above. Normalized data to the individual HGB values on day 0; mean ± SD. (**C**) Representative CD31 immunohistochemistry of femoral bone marrow from a ponatinib-treated mouse and a vehicle (citrate buffer) control mouse culled at the same disease timepoint. Scale bar, 100 µm.

In a translational approach, we applied a widely used animal model of MM [25], in which 5T33 myeloma cells expressing mutant FGFR3 [26] were injected. Ponatinib-treated C57BL6/KaLwRij mice showed significantly prolonged survival in response to administration of ponatinib with a log-rank *p*-value of 0.0065 (Figure 5A). The median survival was 32 days (95% CI 29–33) in the control group and 37 days (95% CI 32–40). The hazard ratio was 0.42 (95% CI 0.23–0.79), indicating ponatinib-treated mice had a 58% lower hazard of death (Mantel–Haenszel method). Of note, no hematological toxicity was observed, and there was a statistically non-significant trend towards higher hemoglobin (HGB) levels in ponatinib-treated animals, likely reflecting a lower myeloma burden (Figure 5B). In these animals, ponatinib treatment led to reduced neo-angiogenesis in the bone marrow, a hallmark of myeloma progression, represented by lower CD31 expression in the stromal cells (Figure 5C).

## 3. Discussion

Personalized oncology has transformed treatment in hematological malignancies by targeting activating mutations and translocations with tyrosine kinase inhibitors [27]. In MM, targeted therapy with antibodies against surface proteins, such as SLAMF7 (signaling lymphocyte activation molecule family member 7), BCMA (B-cell maturation antigen), and GPRC5D (G protein-coupled receptor class C group 5 member D), has significantly improved patients’ prognosis [15,16,17,28]. Moreover, the first attempts at personalized treatment for MM patients with B-Raf proto-oncogene (*BRAF*) V600E mutation [29] or t(11;14) [30] were promising.

Although the prognosis of patients harboring t(4;14), especially combined with chromosome 1q alterations [31], is dismal, this has not yet led to tailored therapy. Previous efforts to target FGRF3 in myeloma with a small molecule inhibiting FGFR3, PD173074, showed promising results in vitro [18]; however, translational data from a phase 2 study (NCT02952573) investigating the FGFR1-4 inhibitor, erdafitinib, which was completed in 2018, have not been fully published, although a single case with eradication of the myeloma clone harboring the FGFR mutation showed proof of concept [19]. In the same vein, selective FGFR3 inhibitors, such as PD173074 and pemigatinib, did not induce apoptosis as single agents but reduced myeloma-derived IL-6 and VEGF levels in our experiments.

Ponatinib has shown efficacy in treating FGFR-mutated cancer [21], and previous studies identified ponatinib as a potential novel agent in MM treatment. Of note, these studies used a combination of either sirolimus or MEK inhibition to induce apoptosis in a dose-dependent manner [22,23]. In contrast to their data, we showed the single-agent activity of ponatinib. This might be explained by our use of concentrations that were three times higher, and bcr-abl off-target effects depend on higher concentrations [32]. The study by Flietner et al. revealed the higher efficacy of ponatinib treatment in cell lines harboring t(4;14) [22]. Corroborating this, our results indicate a dependence of ponatinib’s single-agent anti-myeloma activity on FGFR3 expression. Ponatinib treatment in U266 cells, a cell line without relevant FGFR3 expression, did not induce apoptosis in our experiments. Our findings in experiments using MM1.S cells underline that at least baseline FGFR3 expression is required to observe substantial effects from ponatinib treatment.

In line with these results, sequencing data revealed that ponatinib treatment in cell lines with t(4;14) and overstimulated FGFR3 pathway (KMS18) and those that lack surface FGFR3 expression (U266) shared only a few differentially regulated genes and pathways. In FGFR3-expressing MM cells, ponatinib altered the expression of genes involved in cell-cycle regulators (*CCND1* and *CCND2*), metabolic and hypoxia-associated genes (*SLC2A3*, *AQP1*, and *HBD*), adhesion and polarity markers (*TNS4* and *CRB2*), and stress-response genes (*TSC22D1* and *BCL2L1*). These genes are highly relevant in functionally high-risk patients [1]. Furthermore, KEGG analysis revealed that pathway alterations in JAK/STAT, PI3K/AKT, TNF, MAPK and NFKB signaling, as well as cell death, such as FoxO, apoptosis and p53 signaling, were dysregulated. These results may indicate that inhibition of FGFR3 signaling and downstream pathways such as JAK/STAT and PI3K/AKT needs to be accompanied by addressing additional pathways affecting cell cycle and cell death. Finally, data obtained in murine experiments showed in vivo activity, which is partly explained by reduced neo-angiogenesis, which is a hallmark feature of MM, resulting in poor clinical outcomes [33]. Angiogenesis is driven by VEGF and IL-6 [34] and was significantly reduced by ponatinib treatment in vitro.

Our data support the therapeutic rationale for targeting FGFR3 in multiple myeloma, particularly in cases harboring the t(4;14) translocation and/or exhibiting hyperactivation of the FGFR3 signaling pathway. However, we demonstrate that myeloma cells lacking FGFR3 expression were intrinsically resistant to this strategy. More critically, while selective FGFR3 inhibition modulated cytokine production, this approach was insufficient to induce apoptosis as a monotherapy. These findings underscore the necessity of combinatorial approaches in the context of personalized treatment strategies for t(4;14)-positive myeloma. FGFR3-directed therapies have to be paired with additional pathway inhibitors to achieve effective induction of cell death, as previous studies have shown that approaches only targeting FGFR3 may not be successful due to clonal heterogeneity in MM. Therefore, further research may unveil novel therapeutic approaches based on this study.

## 4. Materials and Methods

### 4.1. Cell Culture

The murine MM cell line 5T33, cultured in RPMI 1640 + GlutaMAX-I medium (Gibco) supplemented with 10% heat-inactivated fetal bovine serum, 100 U/mL penicillin and 100 µg/mL streptomycin, was used for animal experiments as described below.

For in vitro experiments, the human cell lines KMS18 (RRID:CVCL_A637, purchased from JCRB, Ibaraki, Japan), U266 (RRID:CVCL_0566, purchased from DSMZ, Braunschweig, Germany) and MM.1S (RRID:CVCL_8792, purchased from ATCC, Manassas, VA, USA) were used. Cells were cultured in the abovementioned medium. Ponatinib, PD173074 and pemigatinib were purchased from MedChemExpress (Monmouth Junction, NJ, USA) and used in the indicated concentrations. Dimethylsulfoxide (DMSO) served as a vehicle control. Cells were passaged 24 h prior to use in the experimental setting.

### 4.2. Immunofluorescence

For immunofluorescence staining, MM cells were seeded onto poly-L-lysine-coated coverslips and fixed with 4% paraformaldehyde. Permeabilization was performed with saponin. Antibodies against FGFR3 (RRID:AB_2246903) and phosphorylated FGFR3 (RRID:AB_2103530) were purchased from Cell Signaling Technology (Danvers, MA, USA) or Santa Cruz Biotechnology (Dallas, TX, USA) with corresponding fluorescein isothiocyanate (FITC)-labeled secondary antibodies from Invitrogen. 4′,6-Diamidino-2-phenylindol (DAPI) was used to stain nuclei.

### 4.3. Flow Cytometry

Flow cytometric analysis was performed as previously described. Cells were analyzed on a BD LSRFortessa (BD Biosciences, San Jose, CA, USA). Fluorescent FGFR3 antibody (RRID:AB_11129249) was obtained from R&D Systems (Minneapolis, MN, USA). For apoptosis/necrosis detection, the Annexin V apoptosis detection kit with 7-aminoactinomycin D (7AAD) from BioLegend (San Diego, CA, USA) was used. For the measurement of DNA fragmentation, propidium iodide (PI) staining was performed.

### 4.4. Caspase-3 Activity

Caspase-3 activity was measured using the fluorogenic caspase-3 substrate Ac-DEVD-AMC (Enzo Life Sciences, Farmingdale, NY, USA). The caspase inhibitor Z-VAD-FMK (SelleckChem, Houston, TX, USA) served as a negative control.

### 4.5. Western Blotting

For analysis of protein expression, human MM cell lines were treated with ponatinib as indicated. After 1 h of treatment, whole-cell lysates were generated in radioimmunoprecipitation (RIPA) buffer with phosphatase/protease inhibitors and protein concentration was determined. 20 µg of protein were separated by sodium dodecyl sulfate–polyacrylamide gel electrophoresis (SDS-PAGE) and transferred onto a nitrocellulose membrane. Monoclonal antibodies against FGFR3 (RRID:AB_2246903; AB_2103530), STAT3 (RRID: AB_331588; AB_2491009), STAT5 (AB_2737403; AB_10544692), p38 mitogen-activated protein kinase (p38) (RRID:AB_10999090; AB_331641) or extracellular signal-regulated kinases (ERK) 1/2 (RRID:AB_330744; AB_331646) or their phosphorylated (p-) forms by Santa Cruz Biotechnology or Cell Signaling Technology were applied and protein bands were detected using the WesternBright chemiluminescence kit from Advansta (San Jose, CA, USA).

### 4.6. Cytokine Measurement

Cytokine levels in the supernatant of different myeloma cell cultures were measured using the enzyme-linked immunosorbent assay (ELISA) MAX Deluxe sets for human IL-6 and human vascular endothelial growth factor (VEGF) from BioLegend (San Diego, CA, USA) according to the manufacturer’s instructions.

### 4.7. Animal Experiments

All procedures were approved by the Landesamt für Verbraucherschutz und Ernährung Nordrhein-Westfalen (LAVE, formerly LANUV); Az. 81-02.04.2019.A260. Male and female C57BL6/KaLwRij mice aged 8–12 weeks were used for animal experiments. Mice were injected with 3 × 10^5^ 5T33 cells. After 14 days, animals were gavaged daily with 30 mg/kg ponatinib dissolved in 25 mM citrate buffer or vehicle control. Mice were evaluated daily, and survival was recorded. Blood samples were taken every 7 days and analyzed on a HEMAVET analyzer (Drew Scientific, Dallas, TX, USA). After 50 days, surviving mice were sacrificed. Femora of all animals were further analyzed.

### 4.8. Histological Examination

Femora were decalcified in 10% ethylenediaminetetraacetic acid. CD31 antibody was purchased from Cell Signaling Technology (Danvers, MA, USA). The ZytoChem Plus (HRP, Berlin, Germany) and DAB high-contrast kits (Zytomed, Berlin, Germany) were used according to the manufacturer’s instructions. Nuclei were counterstained with hematoxylin.

### 4.9. RNA Isolation

The Quick-RNA™ Microprep Kit (Zymo Research, Irvine, CA, USA) was used according to the manufacturer’s instructions to harvest RNA from myeloma cells. A Tapestation 4200 (Agilent Technologies, San Jose, CA, USA) was used to control RNA quality.

### 4.10. 3’-mRNA Sequencing

The QuantSeq FWD 3′-mRNA-Seq Kit (Lexogen, Vienna, Austria) was used to prepare libraries. A NovaSeq 6000 platform (Illumina, San Diego, CA, USA) with 1 × 100 bp single-end reads was used for sequencing according to the manufacturer’s instructions, with an average of 10 M raw reads per sample.

### 4.11. Analysis of RNA Sequencing Data

For statistical analysis of RNA sequencing data, we followed the workflow already described elsewhere [35]. Briefly, FastQ files were aligned to the reference genome hg19 and then indexed. Differential gene expression analysis was performed, employing the default settings of DESeq2 (version 1.42.0; RRID:SCR_015687) in R (version 4.3.0) [36,37]. A log2 fold change (FC) in FPKM > 1 (fragments per kilobase per million mapped fragments) and false-discovery rate (FDR) < 0.1 indicated differential gene expression. The web-based tool InteractiVenn (https://www.interactivenn.net/, last accessed 9 December 2025; RRID:SCR_028233) was employed to visualize overlapping and non-overlapping differentially expressed genes (DEGs) between ponatinib-treated U266 and KMS18 cell lines [38]. ClusterProfiler in R version 4.1.2 (RRID:SCR_016884) was used for gene set enrichment analysis [39]. The over-representation of biological processes and pathways within networks was examined using the differentially expressed genes as seeds. The Innate database [40] was used to obtain protein–protein interactions for these genes, which were subsequently analyzed using the web-based tool NetworkAnalyst (https://www.networkanalyst.ca/, last accessed 29 September 2024; RRID:SCR_016909) [41]. Kyoto Encyclopedia of Genes and Genomes (KEGG) (RRID:SCR_012773) pathway analyses are shown.

### 4.12. Statistical Analysis

Except for sequencing studies, data from at least two experiments with 2–4 repeats were pooled and analyzed using Prism (Version 10.1.0, GraphPad; RRID:SCR_002798). For Kaplan–Meier survival curves, survival differences between groups were assessed with the log-rank test, assuming significance at *p*  <  0.05. When comparing more than two groups, a one-way analysis of variance (ANOVA) was applied; otherwise, Student’s t-test was chosen. Significance with a *p*-value below 0.05 was reported as follows: * *p* < 0.05, ** *p* < 0.01, *** *p* < 0.001, **** *p* < 0.0001.

## Figures and Tables

**Table 1:** Ponatinib impairs several pathways in KMS18 cells.

Pathway ID	Pathway Name	Gene Count	Gene Ratio	*p*-Value	FDR
hsa04630	Jak-STAT signaling pathway	19	0.0368	9.83 × 10^−11^	1.56 × 10^−8^
hsa05212	Pancreatic cancer	14	0.0271	6.11 × 10^−11^	1.56 × 10^−8^
hsa05200	Pathways in cancer	33	0.0640	3.26 × 10^−10^	3.46 × 10^−8^
hsa04210	Apoptosis	15	0.0291	2.45 × 10^−8^	1.94 × 10^−6^
hsa04933	AGE-RAGE signaling pathway in diabetic complications	13	0.0252	3.05 × 10^−8^	1.94 × 10^−6^
hsa05220	Chronic myeloid leukemia	11	0.0213	1.20 × 10^−7^	6.35 × 10^−6^
hsa05219	Bladder cancer	8	0.0155	6.39 × 10^−7^	2.90 × 10^−5^
hsa05170	Hepatitis C	14	0.0271	8.93 × 10^−7^	3.55 × 10^−5^
hsa05161	Hepatitis B	14	0.0271	1.64 × 10^−6^	5.24 × 10^−5^
hsa01521	EGFR tyrosine kinase inhibitor resistance	10	0.0194	1.65 × 10^−6^	5.24 × 10^−5^
hsa04151	PI3K-Akt signaling pathway	21	0.0407	1.86 × 10^−6^	5.38 × 10^−5^
hsa05210	Colorectal cancer	10	0.0194	3.63 × 10^−6^	9.61 × 10^−5^
hsa04072	Prolactin signaling pathway	9	0.0174	4.88 × 10^−6^	1.19 × 10^−4^
hsa05202	Transcriptional misregulation in cancer	14	0.0271	7.79 × 10^−6^	1.76 × 10^−4^
hsa05162	Measles	12	0.0233	8.30 × 10^−6^	1.76 × 10^−4^
hsa05166	HTLV-I infection	15	0.0291	1.15 × 10^−5^	2.28 × 10^−4^
hsa05142	Chagas disease (American trypanosomiasis)	10	0.0194	1.85 × 10^−5^	3.34 × 10^−4^
hsa05203	Viral carcinogenesis	14	0.0271	1.89 × 10^−5^	3.34 × 10^−4^
hsa04380	Osteoclast differentiation	11	0.0213	2.24 × 10^−5^	3.75 × 10^−4^
hsa05223	Non-small cell lung cancer	8	0.0155	2.60 × 10^−5^	4.14 × 10^−4^
hsa04068	FoxO signaling pathway	11	0.0213	2.99 × 10^−5^	4.53 × 10^−4^
hsa04218	Cellular senescence	12	0.0233	3.71 × 10^−5^	5.36 × 10^−4^
hsa05145	Toxoplasmosis	10	0.0194	4.16 × 10^−5^	5.75 × 10^−4^
hsa04115	p53 signaling pathway	8	0.0155	4.94 × 10^−5^	6.37 × 10^−4^
hsa05222	Small cell lung cancer	9	0.0174	5.03 × 10^−5^	6.37 × 10^−4^
hsa04919	Thyroid hormone signaling pathway	10	0.0194	5.21 × 10^−5^	6.37 × 10^−4^
hsa05224	Breast cancer	11	0.0213	8.07 × 10^−5^	9.32 × 10^−4^
hsa05169	Epstein-Barr virus infection	13	0.0252	8.20 × 10^−5^	9.32 × 10^−4^
hsa05213	Endometrial cancer	7	0.0136	8.70 × 10^−5^	9.54 × 10^−4^
hsa04390	Hippo signaling pathway	11	0.0213	1.23 × 10^−4^	0.0013
hsa04659	Th17 cell differentiation	9	0.0174	1.51 × 10^−4^	0.0015
hsa05167	Kaposi’s sarcoma-associated herpesvirus infection	12	0.0233	1.59 × 10^−4^	0.0016
hsa04930	Type II diabetes mellitus	6	0.0116	1.83 × 10^−4^	0.0017
hsa04668	TNF signaling pathway	9	0.0174	1.86 × 10^−4^	0.0017
hsa05230	Central carbon metabolism in cancer	7	0.0136	1.81 × 10^−4^	0.0017
hsa05221	Acute myeloid leukemia	7	0.0136	1.99 × 10^−4^	0.0018
hsa05211	Renal cell carcinoma	7	0.0136	2.64 × 10^−4^	0.0022
hsa05144	Malaria	6	0.0116	2.61 × 10^−4^	0.0022
hsa05014	Amyotrophic lateral sclerosis (ALS)	6	0.0116	3.26 × 10^−4^	0.0027
hsa05218	Melanoma	7	0.0136	3.44 × 10^−4^	0.0027
hsa05206	MicroRNAs in cancer	15	0.0291	3.95 × 10^−4^	0.0031
hsa05215	Prostate cancer	8	0.0155	4.04 × 10^−4^	0.0031
hsa01522	Endocrine resistance	8	0.0155	4.34 × 10^−4^	0.0032
hsa04012	ErbB signaling pathway	7	0.0136	9.43 × 10^−4^	0.0068
hsa05164	Influenza A	10	0.0194	0.0010	0.0072
hsa04010	MAPK signaling pathway	14	0.0271	0.0011	0.0074
hsa04152	AMPK signaling pathway	8	0.0155	0.0016	0.0111
hsa05152	Tuberculosis	10	0.0194	0.0017	0.0114
hsa04110	Cell cycle	8	0.0155	0.0020	0.0132
hsa01524	Platinum drug resistance	6	0.0116	0.0022	0.0141
hsa04066	HIF-1 signaling pathway	7	0.0136	0.0024	0.0149
hsa04064	NF-kappa B signaling pathway	7	0.0136	0.0024	0.0149
hsa04931	Insulin resistance	7	0.0136	0.0037	0.0225
hsa05418	Fluid shear stress and atherosclerosis	8	0.0155	0.0041	0.0243
hsa05216	Thyroid cancer	4	0.0078	0.0046	0.0257
hsa00310	Lysine degradation	5	0.0097	0.0045	0.0257
hsa04370	VEGF signaling pathway	5	0.0097	0.0045	0.0257
hsa04071	Sphingolipid signaling pathway	7	0.0136	0.0064	0.0349
hsa03440	Homologous recombination	4	0.0078	0.0067	0.0359
hsa04350	TGF-beta signaling pathway	6	0.0116	0.0070	0.0369
hsa04664	Fc epsilon RI signaling pathway	5	0.0097	0.0083	0.0422
hsa03430	Mismatch repair	3	0.0058	0.0083	0.0422
hsa04060	Cytokine-cytokine receptor interaction	12	0.0233	0.0081	0.0422
hsa04920	Adipocytokine signaling pathway	5	0.0097	0.0088	0.0435
hsa04140	Autophagy-animal	7	0.0136	0.0094	0.0459
hsa04662	B cell receptor signaling pathway	5	0.0097	0.0099	0.0475
hsa04217	Necroptosis	8	0.0155	0.0101	0.0481

KEGG pathway enrichment analysis of DEGs from RNA-seq of KMS18 cells treated with 100 nM ponatinib vs. vehicle control (DMSO). Only significantly deregulated pathways (FDR < 0.05) are shown and sorted by FDR.

## Data Availability

The datasets used and/or analyzed during the current study are available from the corresponding author upon reasonable request.

## Abbreviations

The following abbreviations are used in this manuscript:

MM	Multiple myeloma
FGFR3	Fibroblast growth factor receptor 3
IL-6	Interleukin-6
JAK	Janus kinase
STAT	Signal transducer and activator of transcription
RAF	Rapidly accelerated fibrosarcoma
MAPK	Mitogen-activated protein kinase
PI3K	Phosphatidylinositol 3-kinase
AKT	Protein kinase B
mTOR	Mammalian target of rapamycin
PLCγ	Phospholipase C gamma
PKC	Protein kinase C
IMiD	Immunomodulatory drug
BCMA	B-cell maturation antigen
GPRC5D	G protein-coupled receptor class C group 5 member D
MEK	Mitogen-activated protein kinase kinase
DMSO	Dimethyl sulfoxide
DAPI	4′,6-diamidino-2-phenylindole
FITC	Fluorescein isothiocyanate
7-AAD	7-aminoactinomycin D
PI	Propidium iodide
RIPA	Radioimmunoprecipitation assay buffer
SDS-PAGE	Sodium dodecyl sulfate–polyacrylamide gel electrophoresis
p38	p38 mitogen-activated protein kinase
ERK	Extracellular signal-regulated kinase
p	Phosphorylated
ELISA	Enzyme-linked immunosorbent assay
VEGF	Vascular endothelial growth factor
LAVE/LANUV	Landesamt für Verbraucherschutz und Ernährung Nordrhein-Westfalen (State Office for Consumer Protection and Nutrition of North Rhine-Westphalia)
FPKM	Fragments per kilobase per million mapped fragments
FC	Fold change
FDR	False discovery rate
DEG	Differentially expressed gene
ANOVA	Analysis of variance
KEGG	Kyoto Encyclopedia of Genes and Genomes
TNS4	Tensin 4
AQP1	Aquaporin 1
CCND1	Cyclin D1
APOL4	Apolipoprotein L4
CRB2	Crumbs cell polarity complex component 2
TMEM273	Transmembrane protein 273
FAXDC2	Fatty acid hydroxylase domain containing 2
CCND2	Cyclin D2
SNAI3	Snail family transcriptional repressor 3
TSC22D1	TSC22 domain family member 1
IL10RA	Interleukin-10 receptor subunit alpha
TLCD4	TLC domain containing 4
HBD	Hemoglobin subunit delta
SLC2A3	Solute carrier family 2 member 3
C2orf88	Chromosome 2 open reading frame 88
SNHG15	Small nucleolar RNA host gene 15
TNFRSF10A	TNF receptor superfamily member 10A
METTL7A	Methyltransferase like 7A
BCL2L1	BCL2 like 1
TNF	Tumor necrosis factor
NFKB	Nuclear factor kappa-light-chain-enhancer of activated B cells
HGB	Hemoglobin
SLAMF7	Signaling lymphocyte activation molecule family member 7
BRAFMDPI	B-Raf proto-oncogene
